# Preliminary Effectiveness of a Remotely Monitored Blood Alcohol Concentration Device as Treatment Modality: Protocol for a Randomized Controlled Trial

**DOI:** 10.2196/30186

**Published:** 2022-01-14

**Authors:** Frank D Buono, Colette Gleed, Martin Boldin, Allison Aviles, Natalie Wheeler

**Affiliations:** 1 Department of Psychiatry Yale School of Medicine New Haven, CT United States; 2 Aware Recovery Care Wallingford, CT United States; 3 Omni Institute Denver, CO United States

**Keywords:** alcohol use disorder, wireless breathalyzer, treatment, clinical trial, alcohol abuse, relapse, patient monitoring, mobile app, breathalyzer, sobriety, continuous monitoring, blood alcohol concentration, blood alcohol, alcohol, alcohol use, substance use, adherence

## Abstract

**Background:**

Alcohol use disorder is a chronic disorder with a high likelihood of relapse. The consistent monitoring of blood alcohol concentration through breathalyzers is critical to identifying relapse or misuse. Smartphone apps as a replacement of or in conjunction with breathalyzers have shown limited effectiveness. Yet, there has been little research that has effectively utilized wireless or Wi-Fi–enabled breathalyzers that can accurately, securely, and reliably measure blood alcohol concentration.

**Objective:**

The purpose of this study is to evaluate the impact of a wireless blood alcohol concentration device in collaboration with long-term treatment on dropout rates, psychological distress, treatment motivation, quality of life, and need for higher levels of follow-up care for patients with alcohol use disorder.

**Methods:**

The randomized clinical trial will include two arms, access to the wireless breathalyzer versus no access to the breathalyzer, while both groups have access to treatment. Evaluation will last 3 months with a 6-week follow-up, during which each participant will be interviewed at admission, 1 month in, 2 months in, 3 months in, and follow-up. Individuals will be recruited online through a secure telehealth meeting invitation. Outcomes will focus on the impact of the wireless breathalyzer within the alcohol use disorder population, and the combined effect on psychological distress, treatment motivation, and quality of life. In addition, we intend to investigate the impact of the breathalyzer on dropout rates and participants’ need for higher levels of follow-up care and treatment.

**Results:**

The recruitment of this study started in July 2020 and will run until 2022.

**Conclusions:**

This information will be important to develop cost-effective treatments for alcohol dependence. Ongoing monitoring allows treatment providers to take an individualized disease management approach and facilitates timely intervention by the treatment provider.

**Trial Registration:**

ClinicalTrials.gov NCT04380116; http://clinicaltrials.gov/ct2/show/NCT04380116

**International Registered Report Identifier (IRRID):**

DERR1-10.2196/30186

## Introduction

### Prevalence and Relapse

Alcohol use disorder (AUD) is a chronic disorder that presents with a predisposition to relapse and is among the top 5 leading causes of disability-adjusted life years [1]. Worldwide, 5.9% of deaths (7.6% in men, 4.0% in women) are due to alcohol [2], and within the United States, medical costs for AUD are reported to be over $120 billion per year [3]. In addition, AUD has a high occurrence of other comorbid psychiatric conditions (eg, depression and generalized anxiety) [4,5]. Research has shown that individuals with AUD are more likely to have psychiatric conditions, which can lead to increased treatment delays and poor outcomes [6]. This in turn can exacerbate the psychiatric comorbidity [4,7]. Due to these issues, patients can easily become discouraged during treatment, potentially leading to relapses or setbacks [8] along with reductions in treatment motivation [9]. Palmer et al [10] noted that premature client dropout is exceedingly high (74%) in treatment, hindering potential benefits and preventing successful completion. Moreover, relapse rates for AUD are relatively high, ranging between 30% and 70%, 3 months after treatment [11,12]. Due to early dropout and the likelihood of relapse, recovery should be seen as a continuous process instead of a singular incidence [13], and the consistent monitoring of blood alcohol concentration (BAC) is critical to identifying relapse or misuse.

Standard treatment for those with AUD generally provides a highly structured program of care [8,14]. Regardless of the structure of care (group home, step-down approach, individual treatment, or within-home treatment modalities), it is critical to ensure ongoing monitoring by treatment providers to facilitate appropriate patient care and evaluation [15]. In attempting to capture alcohol usage through self-report, many issues can arise, such as lack of recall and deception. To combat these issues, the use of technology via mobile phones, video recording, and behavioral contracts has been utilized [16-18]. It should be noted that Gustafson et al [17] have demonstrated the initial effectiveness of using a smartphone app during recovery in reducing the incidence of hazardous drinking and promoting abstinence. However, a recent systematic review of mobile apps for AUD, showed limited effectiveness when comparing to controlled samples [19]. Devices that can be attached to mobile phones to evaluate BAC have been less than reliable and lack consistency in reporting. There is little research that effectively utilizes wireless or Wi-Fi–enabled breathalyzers that can accurately, securely, and reliably measure BAC [20].

### Effectiveness of Breathalyzers

Breathalyzers have been utilized as a reliable method for detecting the presence of alcohol for over 50 years [16,21]. By collecting real time information, the breathalyzer allows for in vivo evaluation, providing the clinician, researcher, and patient further understanding of usage. Wearable monitors have been shown to be reliable in producing alcohol values [22,23]; however, breathalyzers are less intrusive than wearable monitors as they do not need to be worn, the process is automated, and they encourage accountability via physical exertion (eg, by manually blowing into the device). Previous research has incorporated breathalyzers to ensure that other treatment strategies (eg, contingency management) have been utilized correctly [24]. The authors of the previous study utilized a breathalyzer distributed by Soberlink (Soberlink Healthcare LLC) [24]. Yet, to the best of our knowledge, no randomized clinical trial has been conducted utilizing the breathalyzer in conjunction with the treatment modality, while comparing the absence of the breathalyzer. For that reason, having a device such as Soberlink, which can be utilized as a treatment assessment for the clinician and self-monitoring device for the patient, is critical for treatment centers that take care of individuals with AUD. Building off the research of behavioral psychology [25] and the concept of permanent product, we anticipate that the physical presence of the device will provide accountability, reminder, and reinforcement, which will reduce the likelihood of dropout.

### Objectives and Specific Aims

The purpose of this study is to evaluate the usage and impact of the Soberlink BAC device in collaboration with Aware treatment (receiving AUD treatment through Aware Recovery Care) for patients with AUD. The three main objectives are as follows:

1. To determine the initial impact of the Soberlink device in conjunction with the Aware Treatment model to decrease dropout rates for individuals suffering from AUD.

2. To evaluate the combined effect of Soberlink and Aware Treatment on participants’ treatment motivation, quality of life, and psychological distress.

3. To evaluate the extent to which the need for higher levels of follow-up care and treatment is reduced for individuals who have had access to the Soberlink device and Aware Treatment.

## Methods

### Participant Recruitment, Enrollment, and Randomization

Admission staff at Aware Recovery Care will inform all eligible patients about the research opportunity. Admissions staff will direct patients to flyers or provide the contact information of the principal investigator to set up a meeting, if desired. Flyers with the name and telephone number of the principal investigator will be hung around the main facility and breakout rooms where potential participants congregate. The participant inclusion criteria are as follows: (1) they are at least 21 years old; (2) they are currently enrolled with Aware Recovery Care in-home treatment; and (3) they have a primary or secondary *DSM-5* (*Diagnostic and Statistical Manual of Mental Disorders*) diagnosis of AUD. Previous research by Brown et al [26] demonstrated that late adolescence (16-20 years) is still considered a developmental stage and has heighted challenges with treatment during this age bracket. The exclusion criteria were as follows: (1) having a current suicide or homicide risk, as defined by the SCID-5 (Structured Clinical Interview for *DSM-5*); (2) meeting the criteria for *DSM-IV* current psychotic disorder or bipolar disorder (previously diagnosed); (3) not having phone access with text message capabilities; (4) being unable to read or understand English; (5) being unable to complete the study because of anticipated incarceration or move; (6) having life-threatening or unstable medical problems; (7) having current or pending legal action; and (8) Soberlink results being used for child custody or legal circumstance.

Eligibility will be evaluated by the research assistant and reviewed with the project director or principal investigator prior to initiation. All eligible participants are required to be recruited within 72 hours of being admitted to the program. The participants will be interviewed via Zoom using standardized psychological assessments or will complete similar standardized self-report forms. This is a closed, web-based online recruitment in which quasi-anonymous measures are provided to the participants. For all participants, the principal investigator or a research assistant will meet with them to read through the informed consent document. Each participant will be asked several times throughout the consent procedure whether they understand the information presented and implications for participation. In addition, the subjects will be asked to provide a summary of their understanding of the consent. The participants will also be asked what questions they have about the study procedures, their rights and obligations, and the expectations and obligations of the study staff. When it is clear that the participants understand the material, both the research staff and the participants will electronically sign the consent form.

**Figure 1:**
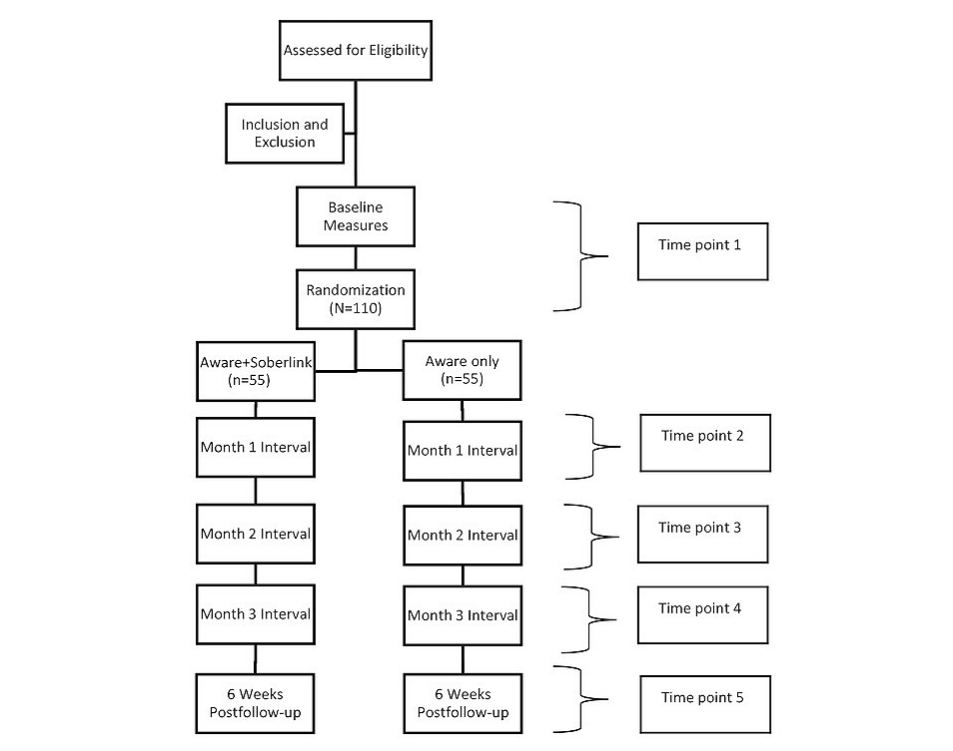
CONSORT (The Consolidated Standards of Reporting Trials) study diagram.

The study will utilize a parallel randomized controlled trial design including 2 groups (Aware treatment only versus Soberlink plus Aware treatment), in which the Aware treatment only group will act as an active control group (a further detailed explanation of the groups is described below). The block randomization sequence will allow for equal representation between the 2 groups without bias on gender, age, or ethnicity, given that all individuals will be categorized as a single variable [27]. The intervention for both groups will last for 3 months with a 6-week follow-up, during which each participant will be interviewed at admission (T1), 1 month in (T2), 2 months in (T3), 3 months in (T4), and follow-up (T5), as seen in Figure 1 [28]. This study has been approved by the FB human research protection program and is registered at ClinicalTrials.gov (NCT04380116).

### Procedure

We are planning to assess a broad range of subject characteristics, process measures, and treatment outcomes over the 3-month trial and at the 6-week posttreatment follow-up, in which all assessments are self-reported and completed online. Baseline assessments are designed to ensure that patients meet eligibility criteria and that important predictor variables such as cognitive impairment are assessed. Baseline measures include evaluation and understanding of the extent of the AUD issue from several different vantage points. Primary outcome measures include reductions in days per week of use and negative BAC screens for alcohol. Monthly assessments will take approximately 30 minutes to complete. Table 1 provides a complete description of the assessments utilized in this study.

**Table 1:** Description of measures.

Measure and description				Completion time and admission										
				Time to complete		T1^a^		T2^b^		T3^c^		T4^d^		T5^e^
**Screening and background measures**														
	Patient Health Questionnaire (PHQ)	A 9-item self-report scale to evaluate depression, which scores each of the 9 *DSM-5*^f^ criteria.	2-3 mins		X		X		X		X		X	
	Generalized Anxiety Disorder Scale (GAD-7)	A 7-item self-report scale used for screening, diagnosis, and severity assessment of anxiety disorder.	2-3 mins		X		X		X		X		X	
	Structured Clinical Interview for *DSM-5* (SCID-5)	A semi-structured interview used to determine *DSM-5* diagnoses.	5 mins		X		N/A^g^		N/A		N/A		N/A	
	Background questionnaire	This questionnaire will assess sociodemographic variables, pain location, number of surgeries, current medications, and access, use, or comfort with smartphones and internet technology.	5 mins		X		N/A		N/A		N/A		N/A	
**Process measures and treatment outcomes**														
	Alcohol Use Disorder Identification Test (AUDIT-C)	A 3-item scale used to determine alcohol use disorder or alcohol dependence.	1-3 mins		X		X		X		X		X	
	Soberlink’s engagement	Application usage (eg, login, dropout rates, compliance, and missed tests) at the user and aggregate level.	N/A		X		X		X		X		X	
	Quality of Life Scale (QOLS)	A 16-item self-report scale used to assess quality of life based on 5 domains of life (material and physical well-being, relationship with other people, social, community and civic activities, personal development and fulfillment, and recreation).	5 mins		X		X		X		X		X	
	Alcohol Abstinence Self-Efficacy Scale	A 40-item self-report scale with 4 subscales (negative affect, social/positive, physical and other concerns, and cravings and urges) used to determine the ability to avoid drinking in different situations based on temptation and confidence.	5-10 mins		X		X		X		X		X	
	University of Rhode Island Change Assessment Scale (URICA)	A 32-item self-report scale with 4 subscales (precontemplation, contemplation, action, and maintenance) used to assess stages of change relating to alcohol use.	5-10 mins		X		X		X		X		X	
	Timeline Follow Back^h^	An instrument used to obtain estimates of the amount of substance used over a period ranging from 30 days to 1 year prior to assessment.	Varies upon interval		X		X		X		X		X	

^a^At admission.

^b^After 1 month.

^c^After 2 months.

^d^After 3 months.

^e^At follow-up.

^f^*DSM*: *Diagnostic and Statistical Manual of Mental Disorders*.

^g^N/A: not applicable.

^h^Permission to utilize granted by Mark and Linda Sobell.

#### Aware Treatment

All individuals will have access to the Aware Recovery Care in-home treatment model, which is a 4-phase step-down model that provides access to continual care through an integrated team of medical, nursing, and home health advisors, as well as counselors coming to the patient’s home [29,30]. Aware Recovery Care is an in-home addiction treatment program that provides highly intensive, rigorously monitored, intensive outpatient care, based on a chronic disease model of care. The approach implements principles of evidence-based practices including motivational interviewing, cognitive behavioral therapy, and dialectical behavioral therapy. Additionally, all families are offered education about AUD, the opportunity to participate in the treatment process, and frequent communication about treatment progress. The patient’s individualized treatment team consists of 1 licensed professional (nurse or master’s level clinician), 2 certified recovery advisors, and 1 licensed marriage and family therapist. It should be noted that due to COVID-19 restrictions, a majority of sessions are provided remotely through telehealth platforms, instead of in-person.

The patient’s care team is overseen by a master’s level alcohol and drug counselor, an advanced practicing nurse, and an addiction psychiatrist. The licensed professional is responsible for leading the team, collaborating with external providers, communicating with family members, and providing referrals to external providers as needed. The certified recovery advisors are lived-experience advisors, at least 1 of whom is both age- and gender-matched in order to provide a more intimate therapeutic relationship. Certified recovery advisors are responsible for delivering a staged biopsychosocial curriculum. All care teams include 1 licensed marriage and family therapist for 4 family therapy sessions, as well as 4 family education sessions delivered by 1 specially trained family education facilitator.

#### The Aware Plus Soberlink Group

This group will include access to the Aware treatment and to the Soberlink device. During enrollment, a research assistant will train the participant and provide an orientation to use the Soberlink device (see “Orientation to Soberlink” below). All participants will complete screening and outcome assessments, along with using the Soberlink device for the first time. The participants’ Soberlink device will be set up to test them for alcohol use twice a day. Reminder emails will be sent out the day before and on the day of the 1-month, 2-month, and 3-month surveys. All surveys will be completed online in the participant’s home through a secure link sent directly to them. Subsequent phone calls will be made by the research assistant to the participant if they have missed their evaluation. Upon completion of the surveys, the participants in the Aware plus Soberlink group will be paid and reminded of the subsequent follow-ups. The estimated time of the consent process will take 45 to 60 minutes to complete.

#### Orientation to Soberlink

Patients assigned to the Aware plus Soberlink condition will receive a 30-minute orientation to the Soberlink device, including an initial measurement of their BAC. The Soberlink device consists of a wireless breathalyzer that uses a professional-grade fuel cell sensor to detect alcohol levels at an accuracy of +/-0.005 BAC. The device also has an embedded camera and uses facial recognition software to automatically identify the patient during the test. The participant will have a set testing schedule that consists of 2 tests per day. The participant will have a test window of 2 hours with a late window of 1 hour. When the participant blows into the Soberlink device, it will capture the BAC level while the embedded camera takes a photo of the participant during the test. A visual indicator on the Soberlink device or in the Soberlink app will display “compliant” or “non-compliant” at the time of examination.

The breathalyzer uses wireless technology to send the data in real time. The cloud-based monitoring system includes a scheduler that automatically tracks scheduled tests and sends reminders to the patient for testing. Retesting is automatically scheduled if a positive test is received or if a patient’s identity cannot be verified. The device locks out for 15 minutes to ensure alcohol evaporates before the next test. The facial recognition software ensures identity is approved or declined in real time. Artificial intelligence is used to report on testing, and green, yellow, and red visual icons are used in all reporting to identify events.

A testing window will be scheduled with everyone, between 7 AM and 9 AM (morning BAC) and from 8 PM to 10 PM (evening BAC). Individuals in this group can provide the BAC at any time within the testing window. All tests performed outside the window will be recorded but counted as unscheduled tests on the BAC spreadsheet. Previous research has used fixed windows for evaluation. These windows allow for structure in treatment for the individual, reducing the need to always carry the device on one’s person [31]. All participants can opt in to receive texts from Soberlink to remind them about their morning and evening BACs.

#### Aware Group

This group will not receive access or training to a Soberlink device; however, they will be recruited to complete the same set of surveys at the same time intervals (Figure 1). Reminder emails will be sent out the day before, and on the day of the 1-month, 2-month, and 3-month evaluations.

### Retention and Adherence

We intend to over-recruit participants by 25% to account for attrition and potential dropouts. In substance use disorder research, it is reported that 73% will drop out of treatment within the first 3 months due to noncompliance of treatment [10]. All participants will be compensated $10 for the baseline and monthly assessments and $25 for follow-up assessment (up to $65 total). Soberlink will purchase gift cards and the principal investigator or research assistant will distribute them to the participants. Patients who are randomized to the Soberlink group will receive the Soberlink device and 4 months of Soberlink service for no charge. To control for attrition and increase compliance with the device, the following protocol will be put into place. If individuals miss 2 consecutive BACs, participant’s case managers will be informed by the research assistant or the principal investigator. If an individual misses 4 consecutive BACs without communication, it will result in termination from the study by the principal investigator. Eliminating participants who are not adhering to the research design is a critical element for a successful trial [32,33]. This will reduce the likelihood of those reporting clinical improvement regardless of actual response, while establishing systematic methods to detect and eliminate nonadherence, which is in line with the method utilized by Shiovitz et al [33].

### Sample Size

A sample size of 110 would provide >80% power (α=.05) to detect a medium-to-large effect size (*d*=0.65) and allow to fully evaluate gender differences. Given this, we expect to enroll 145 participants into the study. Both groups should have even distribution (n=55) within each group and estimated equal differences between genders (n=25), allowing up to 5 to be transgender or “not to specify” [34].

### Data Analysis

Statistical procedures and models for analyzing data have been selected according to the research hypotheses being investigated and the types of data available. We will use α<.05 but will use appropriate corrections for multiple tests. Statistical analyses will be conducted using SPSS (IBM Corp). If missing data occurs from participants who have completed the time intervals, we intend to use missing values analysis prior to running any analysis. The missing values analysis is the most conservative measure, as described by Tabachnick and Fidell [35], to detect any missing values. If more than 5% of the data are missing for a specific assessment, the data will be eliminated from the data set to reduce potential errors and increase of bias.

To understand the first aim, a repeated measures general estimating equation and linear mixed model analysis for the primary outcomes of negative BAC and days or months of self-reported alcohol use will be conducted. This will be used to determine the initial impact of the Soberlink device in conjunction with the in-home addiction treatment model, to evaluate the combined effects, and to assess the need for higher levels of follow-up. Sample size calculations for linear mixed model analysis are dependent on the number of repeated events, the retention rate, and the interclass correlation.

For the second and third aims, chi-square and *t* tests will be used to conduct preliminary analyses of the adequacy of the randomization procedure, the comparability of baseline measures for the 2 groups, and the possible need for covariates in the analyses of treatment outcome data. Repeated-measures ANOVA will be used to evaluate differences in process measures acquisition over time across each grouping. Additionally, the Pearson product-moment correlations will be used to assess the relationship between process measures and active use based on the Soberlink device testing results over the duration of the study.

## Results

Recruitment of this study started in July of 2020 and will run until 2022. Enrollment has been slower than anticipated due to the COVID-19 pandemic. This study is expected to conclude on July 1, 2022.

## Discussion

This study will assist in evaluating the efficacy of the combined effect of the Soberlink device and Aware Recovery Care model. The study is categorized as “minimal risk,” given the limited risks it has (risks are commensurate with everyday risks associated with alcohol abuse treatment as an adequate protection of confidentiality). This information is important in order to develop cost-effective treatments for alcohol dependence, which ultimately would be of great benefit to the society [14]. Considering the anticipated benefits of the study to the subjects and to society, the low risks it poses to the subjects are reasonable. For many patients, AUD is a chronic medical condition characterized by multiple periods of relapse and sobriety. Much like with the management of other chronic diseases, patients with AUD often seek out treatment multiple times to manage the symptoms of their disease [12]. To effectively treat AUD as a chronic disease, it is critical to regularly monitor key indicators of disease control such as BAC. Ongoing monitoring allows treatment providers to take an individualized disease management approach and facilitates timely intervention by the treatment provider. The Soberlink device automatically notifies treatment providers of positive BAC tests in real time. After being notified, treatment providers can respond quickly and determine if changes are necessary to a patient’s treatment plan or if the patient should be moved to a higher level of care. In addition to intervening after a positive Soberlink test, multiple missed tests by a patient can also provide valuable information to a treatment provider about a patient’s level of engagement and signal the need for outreach.

In this study, the Soberlink device is being used by patients receiving outpatient treatment through Aware Recovery Care. However, the device may be useful to patients with AUD in a variety of settings, across multiple phases of recovery. The device serves as an ongoing source of connection and point of engagement with a treatment provider for patients during treatment and beyond discharge. Using the Soberlink device may provide AUD patients with both personal and social accountability. Patients may also find that the device serves as an external source to support self-control, and those that choose to share results with family members may find that it helps to rebuild trust that may have been lost over time. In addition to reducing alcohol use, using the Soberlink device may positively impact other whole health outcomes such as improved relationships, employment or school engagement, management of health and stress, and reduction of mental health symptoms.

## Abbreviations

AUD: alcohol use disorder
BAC: blood alcohol concentration
*DSM*: *Diagnostic and Statistical Manual of Mental Disorders*
SCID: Structured Clinical Interview for *DSM*
